# Earnings Quality Measures and Excess Returns

**DOI:** 10.1111/jbfa.12071

**Published:** 2014-04-19

**Authors:** Pietro Perotti, Alfred Wagenhofer

**Keywords:** earnings quality, excess returns, abnormal returns, accruals quality, smoothing, value relevance

## Abstract

This paper examines how commonly used earnings quality measures fulfill a key objective of financial reporting, i.e., improving decision usefulness for investors. We propose a stock-price-based measure for assessing the quality of earnings quality measures. We predict that firms with higher earnings quality will be less mispriced than other firms. Mispricing is measured by the difference of the mean absolute excess returns of portfolios formed on high and low values of a measure. We examine persistence, predictability, two measures of smoothness, abnormal accruals, accruals quality, earnings response coefficient and value relevance. For a large sample of US non-financial firms over the period 1988–2007, we show that all measures except for smoothness are negatively associated with absolute excess returns, suggesting that smoothness is generally a favorable attribute of earnings. Accruals measures generate the largest spread in absolute excess returns, followed by smoothness and market-based measures. These results lend support to the widespread use of accruals measures as overall measures of earnings quality in the literature.

## 1. INTRODUCTION

There has been considerable interest in measuring the quality of financial reporting. For example, some empirical studies analyze earnings quality trends over time and their determinants; others measure the effects of specific changes in accounting standards, enforcement systems, or corporate governance requirements within or across countries. Further studies use earnings quality to explain variations in economic outcomes, such as the cost of capital. Since earnings quality is not directly observable, the literature has developed a variety of proxies for earnings quality (surveyed in, for example, Schipper and Vincent, 2003; Dechow and Schrand, 2004; Francis et al., 2006; and Dechow et al., 2010). Most of them are based on intuitive and plausible concepts about desirable characteristics of an accounting system. Although the measures are intended to capture the same fundamental construct, they correlate only weakly. This makes the question of which measure to use a critical research design issue, and is likely to have a significant effect on the results. Unfortunately, there is little guidance as to how good the proxies for earnings quality really are and what a best measure in any given circumstances might be.

A ranking of earnings quality measures requires a measure of the quality of earnings quality measures. This paper builds on a fundamental objective of financial reporting, which is the usefulness of reporting to investors in making resource allocation decisions. It proposes a security-based measure: we predict that the stocks of firms with higher (true) earnings quality will be less mispriced than those of other firms. Mispricing due to poor earnings quality increases errors in pricing firms, but does not lead to systematic under or overpricing. Therefore, we measure mispricing by the firm-specific absolute value of excess returns. An earnings quality measure is of higher quality than another if it better explains variations in absolute excess returns. To test this, we form portfolios of firms with high and low values of earnings quality measures and calculate the difference of the mean absolute excess returns. A larger difference indicates a better earnings quality measure.

We consider four sets of measures with two commonly used measures each: time-series measures (persistence, predictability), smoothness measures, accruals measures (abnormal accruals, accruals quality), and value relevance measures. We perform our tests on a large sample of US non-financial firms over the period 1988–2007. First, we find that time-series measures, accruals measures (multiplied by –1) and market-based measures are negatively associated with absolute excess returns; this is consistent with the use of these measures in the literature. However, we also find that smoothness measures are positively associated with absolute excess returns, which is in contrast to the prevailing interpretation of smoothness as having a negative impact on earnings quality. Our main finding is that accruals quality best discriminates between the absolute values of excess returns, with abnormal accruals as second-best measure. A possible reason that both accruals measures do better than the other measures is that they utilize more information. Next best are smoothness measures and the earnings response coefficient, predictability and persistence do significantly less well, and value relevance brings up the rear. These findings lend support to the widespread use of accruals measures in the empirical earnings quality literature.

We test for alternative explanations for excess returns, including information uncertainty, innate factors, and the information environment, and we vary the determination of expected returns. Our results are also robust to a variety of sensitivity tests. However, while we find robust evidence in support of our hypotheses, we cannot completely exclude other possible explanations for a relationship between earnings quality measures and excess returns. We perform several analyses to control for other explanations and find that our results are not significantly affected.

We are not aware of other literature that explicitly ranks earnings quality measures with respect to their effect on better investor decision making. There is little theoretical literature addressing this issue, and it produces mixed results (see, for example, Ewert and Wagenhofer, 2012). This literature is based on rational market pricing, defining away potential mispricing. Few empirical studies compare a broad set of earnings quality measures. For example, Francis et al. (2004) study seven earnings quality measures and their association with cost of equity capital and realized returns. They find that accounting-based measures, in particular accruals quality, are more strongly associated with their measures of cost of capital than market-based measures.

Our approach is related to the literature on earnings quality and abnormal trading profitability. This literature typically uses a hedge returns approach, which is based on *signed* excess returns. Several papers, for example, Aboody et al. (2005), Mashruwala and Mashruwala (2011), Shi and Zhang (2011), Ogneva (2012) and Brousseau and Gu (2012), study trading strategies based on accruals measures of earnings quality. Other papers, such as Francis et al. (2005), Core et al. (2008), examine abnormal trading profitability by investigating whether earnings quality is a priced risk factor. Hedge strategies aim at exploiting systematic market mispricing effects. In most cases, they are based on the prediction that investors under-react to available information. In contrast, our paper does not predict a systematic biased reaction, but focuses on the spread of reactions, i.e., a second-moment rather than a first-moment effect. Another set of papers, beginning with Xie (2001), investigate the mispricing of signed discretionary accruals. For example, Cheng et al. (2012) examine the performance of abnormal accrual models in predicting 1-year-ahead returns as a proxy of mispricing, and identify the modified Jones model with operating cash flows as having the strongest association with returns.

Our analysis also draws on the literature on the general relationship between information uncertainty and market mispricing. Easley and O'Hara (2004) provide a basis for a large part of this stream of research. Proxies for information uncertainty typically include accounting and market data, such as cash flow volatility, firm age, return volatility and analyst-forecast properties. One set of papers tests whether information uncertainty is associated with underpricing or overpricing of stocks. For example, Diether et al. (2002) and Jiang et al. (2005) find that information uncertainty is related to overpricing. Berkman et al. (2009) report that firms with greater information uncertainty experience lower stock returns around the time of earnings announcements. Other studies are concerned with the association between information uncertainty and known mispricing patterns. For example, Zhang (2006) finds that information uncertainty is positively associated with price under-reaction to public information. Francis et al. (2007) study the relationship between information uncertainty and post-earnings-announcement drift. We predict no particular direction of mispricing due to differences in earnings quality and do not condition our analysis on specific events, because the earnings quality measures we use are based on annual financial information.

Finally, our paper is related to the literature on the relationship between earnings quality and idiosyncratic stock return volatility.^1^ For example, Rajgopal and Venkatachalam (2011) find a positive association between earnings quality, measured in terms of accruals quality and abnormal accruals, and idiosyncratic return volatility. Chen et al. (2012) document a positive association between the absolute value of discretionary accruals and idiosyncratic volatility. Hutton et al. (2009) find that financial statement opacity, measured using discretionary accruals, is negatively associated with idiosyncratic volatility. Unlike these papers, our analysis focuses on the effects of different earnings quality measures, and considers long-term effects of varying earnings quality.

The paper proceeds as follows: in the next section, we develop the main argument and formulate the hypotheses. Section 3 explains our research design, including how the measures and excess returns are calculated. Section 4 describes the sample. Section 5 contains the results of the empirical tests, including alternative explanations and sensitivity tests. Section 6 provides a summary conclusion.

## 2. HYPOTHESES DEVELOPMENT

### (i) Measures of Earnings Quality

Earnings quality is a key characteristic of financial reporting. It embodies the principle that financial reports should be as useful as possible to investors and other capital providers in making their resource allocation decisions. High-quality financial reports should improve decision making and, thus, capital market efficiency. Earnings quality is however an elusive construct and people tend to understand it in various different ways. There is no generally accepted measure, but the literature has developed a variety of proxies for earnings quality, which focus on particular attributes of what earnings quality is considered to be. In this paper we examine how these measures fulfill the objective of improving the decision usefulness of financial reports, i.e., how good their quality is.

We select eight earnings quality (EQ) measures that are commonly used in the empirical literature. They include accounting-based and market-based measures. Accounting-based measures only use accounting earnings and components thereof, whereas market-based measures are based on accounting earnings and market returns. Within the group of accounting-based EQ measures we consider measures that are based on the time series of earnings, on their volatility or smoothness, and on the unexpected part of accounting accruals.

Our time-series measures are persistence and predictability. Persistence measures the extent that current earnings persist or recur in the future. High persistence is positively associated with high earnings quality, since it indicates a stable, sustainable and less volatile earnings generation process that is particularly valued by investors. Predictability captures the notion that earnings are of higher quality the more useful they are in predicting future earnings. Similar to persistence, predictability is viewed as a desirable attribute of earnings because it increases the precision of earnings forecasts. The time series of earnings is affected by the volatility of operations, the economic environment and the accounting systems employed.

The second set of EQ measures reflects smoothness of earnings. We use two smoothness measures based on the volatility of earnings or accruals relative to the volatility of operating cash flows. These measures use operating cash flows as the reference proxy for performance, which presupposes that cash flows are not subject to earnings management. Earnings smoothness has been used differently in empirical studies. A common view is that smoothness is negatively associated with earnings quality (e.g., Nanda et al., 2003). In this view, smoothness is considered to be a consequence of earnings management, i.e., deliberate smoothing by managers. Earnings management is an attempt to mask a firm's “true” performance, and reduces the information value of reported earnings, making them less useful. An alternative view starts with the observation that the objective of accounting is to determine earnings, which are operating cash flows plus accounting accruals, and that the purpose of accruals is to smooth cash flows by filtering out some of their volatility. In a similar way to persistence and predictability, a smoother earnings stream is less volatile and makes better forecasting possible. Some smoothing must therefore be desirable, otherwise users would simply consider cash flows and ignore earnings. Moreover, since management uses its private information to decide on the amount of bias, smoothing incorporates private information about future cash flows into concurrent earnings (“forward” smoothing). Prior studies provide evidence that practitioners (Graham et al., 2005) and investors (Allayanis et al., 2008) view smoothness as a desirable attribute of earnings. Under this alternative view, smoothness should be positively associated with earnings quality.

The third set of earnings quality measures focuses on accruals. One common approach is to split accruals into “normal” and “abnormal” accruals, based on a forecast model for total accruals (following Jones, 1991). Abnormal accruals are the difference between actual and expected accruals. Higher (absolute) abnormal accruals are commonly interpreted as meaning lower earnings quality, because the firm's accrual process is less predictable and abnormal accruals are likely to be discretionary, i.e., from the result of earnings management. The alternative view here is that abnormal accruals are the means within the accounting system of communicating private information. Abnormal accruals are thus an indicator of high earnings quality, although the effect is reduced by any deliberate earnings management. Rational expectations market models suggest that the information component outweighs the earnings management component, because rational investors use their knowledge about management incentives to remove the expected earnings management component from reported earnings. The amount of abnormal accruals does not capture this potential market reaction, and therefore abnormal accruals should be a less useful proxy for earnings quality.

A second common accruals measure is accruals quality (Dechow and Dichev, 2002). This measure maps working capital accruals to lagged, contemporaneous and future cash flows from operations. According to prior literature, the better this mapping explains the accruals, the lower is the residual from a regression based on these cash flows and the higher is the earnings quality. The empirical literature suggests accruals quality is a better measure than other accounting-based measures, and therefore it is used in many studies. In addition to its economic appeal, a reason for its superiority may stem from the fact that accruals quality includes one-period-ahead cash flows and, thus, more information than the other measures. However, accruals quality is subject to concerns similar to those noted for abnormal accruals, as the residual reflects not only earnings management but also potentially useful firm information.

The most common of the market-based measures is value relevance. This is measured by the earnings response coefficient, which is the slope coefficient in a regression of the market returns on earnings, sometimes augmented by changes in earnings, or by the *R* ^2^ of such a regression. High value relevance is generally considered to indicate high earnings quality. Although there is concern about inferences one can draw from value relevance studies (see, e.g., Holthausen and Watts, 2001), this concern comes more from the contracting role of accounting than from the decision-usefulness approach that underlies financial reporting standards (e.g., FASB, 2010).

### (ii) Excess Returns and Earnings Quality

We propose a stock-price-based measure for assessing the quality of earnings quality measures. A major objective of accounting information is to provide investors with information to enable them to make optimal capital allocation decisions, so that stock prices aggregate financial information and other information available in capital markets efficiently. Our measure is based on the association between the earnings quality measures and the absolute value of future excess returns. Excess returns are defined as the difference between actual returns and expected future returns. Given an appropriate model for determining expected future returns, excess returns arise for two reasons: one is the inherent uncertainty of the profitability of firms’ operations (shocks) and the other is market mispricing. We have no reason to expect that earnings quality is systematically associated with unexpected shocks to operating profitability. However, market mispricing should systematically vary with earnings quality measures because better financial information should reduce mispricing.

We predict that earnings quality will affect the level of mispricing in the following way: firms that report financial information with higher earnings quality provide more transparent and precise information to investors. Investors use this superior information to price the firms, resulting in less mispricing than for firms with lower earnings quality. Poor earnings quality does not lead to systematic underpricing or overpricing, but to less precise pricing, so a hedge returns design would not be appropriate. Therefore, we use the absolute value of excess returns as the measure of mispricing. This prediction is consistent with findings in Zhang (2006) and Francis et al. (2007), which examine the post-earnings-announcement drift and find that it is stronger for firms with low earnings quality.

There are several reasons for non-directional market mispricing. For example, mispricing can result from behavioral biases of investors, such as different investor degrees of sophistication (Bartov et al., 2000) and limited attention (Hirshleifer et al., 2011). Mispricing can be due to costs of acquiring information (Landsman et al., 2011), transaction costs and limits to arbitrage (Ng et al., 2008; Zhang et al., 2013), divergence of opinions (Garfinkel and Sokobin, 2006) or time of the year (Mashruwala and Mashruwala, 2011). However, Penman and Zhu (2011) find that excess returns can be consistent with rational pricing if earnings and revisions in earnings growth expectations are considered appropriately. Finally, mispricing can also arise because of errors in estimating discount factors; this is the case if uncertainty-averse investors price firms at a discount because they cannot determine the uncertainty.

Absolute excess returns may also be affected by other factors that are aggregated in stock prices. One possibility is that firms exhibiting more fundamental uncertainty have lower earnings quality and higher stock price variability. This association is similar to what we predict where market mispricing occurs. We control for fundamental uncertainty in a number of ways in order to separate this explanation from the mispricing explanation. Low earnings quality of a firm's financial information may also induce investors and financial intermediaries to collect more information, which then reduces the mispricing of such stocks. If other information does not fully substitute for low-quality financial information, it will only lower the power of our tests. Another possible cause of observed mispricing is an inappropriate expected returns model that wrongly identifies excess returns. Moreover, expected returns can vary with earnings quality, although it is not clear how that would affect the association of earnings quality and excess returns. We perform several tests to address alternative causes of a variation of excess returns.

As we discuss in the subsequent section, we calculate the earnings quality measures as common in the literature, but sign them so that a higher measure indicates higher (true) earnings quality. However, smoothness and accruals-based measures can be interpreted in various ways – one view is that higher smoothness or residual accruals indicate earnings management and thus low earnings quality, while the other view is that they provide useful information. Our first hypothesis addresses the question of whether, based on our mispricing measure, an earnings quality measure should be interpreted as positively or negatively associated with earnings quality. Earnings quality is expected to reduce mispricing. Firms that exhibit a higher value of an earnings quality measure are expected to be less mispriced on average. This hypothesis aims to distinguish between these views, and contributes to the debate on whether an earnings quality measure is driven by earnings management or by information communication to market participants. The first hypothesis is:

**H1**: The earnings quality measures are negatively associated with the absolute value of excess returns.

After establishing the existence of an association, we examine how strong the association is for each earnings quality measure. Since true earnings quality implies less mispricing, a measure that shows this effect more strongly should be a more useful measure.

Our second hypothesis is:

**H2**: The earnings quality measures differ in their ability to distinguish firms with high and low earnings quality, as measured by the unsigned difference in their absolute excess returns.

We offer no formal hypotheses on the ranking of earnings quality measures. We note, though, that they are based on different sets of information. Measures that process more information are likely to better discriminate excess returns. Abnormal accruals use the largest set of financial items, accruals quality is based on three subsequent cash flows, and smoothness measures use the volatility or the correlation of cash flows, earnings and accruals. Time-series and value relevance measures use only net income from the financial statements; in addition, value relevance measures rely on market prices. However, as discussed above, accruals and smoothness measures are ambiguous in their interpretation, which is likely to reduce their ability to discriminate excess returns. Hence, the ranking is likely to depend on several, partially countervailing, effects.

The theoretical literature also provides inconclusive results and it assumes rational market pricing, which excludes mispricing. For example, Ewert and Wagenhofer (2012) report studies which find value relevance being most closely related to earnings quality, whereas smoothness and accruals quality do worse. In Marinovic (2013) persistence is a useful measure, whereas predictability and smoothness do not reflect earnings quality. Drymiotes and Hemmer (2013) find that value relevance from a price–earnings regression is an unreliable measure of earnings quality.

Some empirical literature suggests that accruals quality measures, particularly the Dechow and Dichev (2002) measure, are superior measures. Francis et al. (2004) find that accounting-based measures, in particular accruals quality, provide the strongest association with their *ex-ante* cost of capital measure. Our analysis complements their analysis because both expected returns and excess returns are components of realized returns: they focus on the former, while we study the latter. We have no reason to expect that their results should be similar to ours, because the two components are not theoretically linked.^2^

## 3. RESEARCH DESIGN

### (i) Calculation of Earnings Quality Measures

We calculate our eight EQ measures following the literature (e.g., Dechow and Schrand, 2004). A summary description of all variables is given in the Appendix. The base earnings measure is net income before extraordinary items (*NIBE*). Total accruals (*ACC*) is calculated as *ACC* = Δ*CA* – Δ*CL* – Δ*CASH* + Δ*STDEBT* – *DEPR*, where the variables are change in current assets, change in current liabilities, change in cash, change in short-term debt, and depreciation in the fiscal year ending at *t*. Cash flow from operations (*CFO*) is calculated as *CFO* = *NIBE* – *ACC*. Current accruals (*CACC*) is computed as *CACC* = Δ*CA* – Δ*CL* – Δ*CASH* + Δ*STDEBT*. The eight EQ measures are estimated for each firm and year for rolling 10-year periods *t*–9 to *t*. Table1 summarizes the definitions of the EQ measures used.

**Table 1:** Definition of Earnings Quality Measures

*Measure*	*Description*	*Definition*	*Direction of Effect*
Time-series Measures			
EQ1	Persistence	Slope coefficient β from 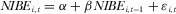	+
EQ2	Predictability	*R* ^2^ from 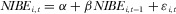	+
Smoothness Measures			
EQ3	Smoothness	Standard deviation ratio 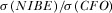	+
EQ4	Smoothness	Correlation 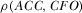	+
Accruals Measures			
EQ5	Abnormal accruals	Negative absolute value of residual from 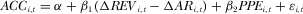	+
EQ6	Accruals quality	Negative standard deviation of residual *ε*_*i*,*t*_ of 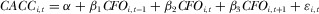	+
Value Relevance Measures			
EQ7	Earnings response coefficient (ERC)	Slope coefficient β from 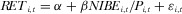	+
EQ8	Value relevance	*R* ^2^ from 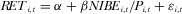	+

*Notes:* This table describes the earnings quality measures used. *NIBE*: net income before extraordinary items; *CFO*: cash flow from operations; *ACC*: total accruals; *CACC*: current accruals; *PPE*: gross property, plant and equipment; *ΔREV*: change in revenues; *ΔAR*: change in accounts receivable. All the aforementioned variables are scaled by total assets at the beginning of the period. *RET*: 12-month stock return ending 3 months after the end of the fiscal year; *P*: market value of equity at the beginning of the fiscal year.

The residual form earnings quality measures are obtained as the residuals – using yearly regressions – of the raw form measures on six innate factors, i.e., *assets*: natural logarithm of total assets; *operating cycle*: natural logarithm of the sum of days accounts receivable and days inventory; *intangible intensity*: reported R&D expense divided by sales; *capital intensity*: net book value of property, plant and equipment divided by total assets; *growth*: percentage change in sales; *leverage*: total liabilities divided by equity book value.

The direction of effect is based on the generally shared view of the association between the value of the measure and earnings quality. For example, a larger value of EQ1 indicates higher earnings quality.

The two time-series measures are persistence and predictability. Persistence (EQ1) is equal to the slope coefficient β of the following regression: (1)
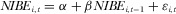
where *NIBE* is scaled by total assets at the beginning of period *t*. Predictability (EQ2) is the *R* ^2^ of this regression.

Our first smoothness measure (EQ3) is the ratio of the standard deviation of earnings over the standard deviation of cash flow from operations, (2)
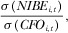
where *NIBE* and *CFO* are scaled by total assets at the beginning of period *t*. The second smoothness measure (EQ4) is based on the correlation of accruals and cash flow from operations, (3)
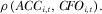
*ACC* and *CFO* are scaled by total assets at the beginning of period *t*. Greater values of EQ3 and EQ4 indicate lower smoothness. Following the interpretation in some of the literature (e.g., Nanda et al., 2003) that views smoothness as an undesirable attribute, Table1 shows a positive sign for EQ3 and EQ4.

Accruals measures are abnormal accruals and accruals quality. Abnormal accruals (EQ5) are estimated based on the following regression:^3^
(4)
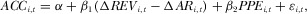
where Δ*REV* is the change in revenues, Δ*AR* the change in accounts receivable, and *PPE* is gross property, plant and equipment. All variables are scaled by total assets at the beginning of period *t*. The abnormal accruals measure is the absolute residuals,^4^ 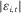, multiplied by negative one.

Accruals quality (EQ6) is based on the residuals of the following regression of current accruals on cash flow from operations: (5)
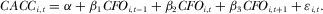


All variables are scaled by total assets at the beginning of period *t*. EQ6 is defined as the standard deviation of the residuals multiplied by negative one. The definition embodies the prevailing interpretation that higher values of EQ5 and EQ6 indicate high earnings quality.

Finally, the two value relevance measures are estimated using the following regression: (6)
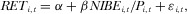
where *RET* denotes the 12-month return ending 3 months after the end of the fiscal year, and *P* is the market value of equity at the beginning of period *t*. Observations with *RET* in the top and bottom one percentile we treat as missing. Our first measure (EQ7) is the earnings response coefficient (*ERC*), which is the β in (6). The second measure (EQ8) is the *R* ^2^ of the regression.

Our estimation of the EQ measures over a rolling 10-year period takes care of industry differences because it uses each firm as its own control. It assumes that earnings quality is a sticky characteristic; accordingly, we also calculate 1-year return windows. As our results indicate, there is still sufficient variability in the hedge portfolios, because firms are assigned to the portfolios based on their relative rather than their absolute earnings quality measures.

### (ii) Calculation of Excess Returns

To compute excess returns, we follow Landsman et al. (2011). We use the three-factor asset pricing model of Fama and French (1993) plus the momentum factor (Carhart, 1997) to estimate the expected risk-adjusted return of each firm in the portfolios.^5^ There may be other common risk factors, but there is no consensus as to which ones are the most descriptive and whether adding additional factors improves the net benefit of forecasting and valuation.

For each firm and month we estimate the factor betas over a 36-month period prior to the respective month by: (7)
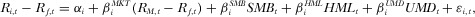
where *R*_*i*,*t*_ is the actual monthly return of firm *i*, *R*_*f*,*t*_ is the monthly riskless rate of return, *R*_*M*,*t*_ is the monthly market return, *SMB*_*t*_ is the monthly return on the size factor mimicking portfolio, *HML*_*t*_ the monthly return on the book-to-market factor mimicking portfolio, and *UMD*_*t*_ the monthly return of the momentum factor mimicking portfolio.

Taking these estimated factor βs for month *t* as expected βs for month *t*+1, we calculate the expected risk-adjusted return from the following equation: (8)
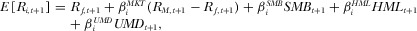
where the factor returns in *t*+1 are obtained as each factor's average monthly return over the previous 36 months.

The excess return of each firm and month is the actual return minus the expected return, (9)
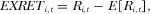
where *EXRET_i,t_* is the month *t* percentage excess return on the stock of firm *i*. Monthly excess returns are then aggregated using 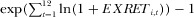 to obtain annual buy and hold returns.

To evaluate the association between the EQ measures and absolute excess returns we use a hedge portfolio approach. This approach is broadly used to assess the trading profitability of hedge strategies and market mispricing.^6^

For each fiscal year we form equal-weighted portfolios of firms for each of the earnings quality measures we study. We do not consider value-weighted portfolios because the results of such portfolios are likely to be driven by a small number of the largest firms. Assuming that financial reports for each year are available within 3 months of the fiscal year end, we start accumulating 12-month excess returns beginning in the fourth month. To avoid concerns about the potential influence of outliers that are likely to be accumulated at the extremes of the distributions, we use quartiles rather than deciles.^7^ The top quartile contains the 25% of firms with the highest value of the earnings quality measure, the bottom quartile the 25% of firms with the lowest value of the earnings quality measure.

We take the difference between the mean absolute excess returns of the firms in the top quartile minus the average of the absolute excess returns of the firms in the bottom quartile as our proxy for the quality of an earnings quality measure and label this difference the absolute excess return spread (AERS). Rebalancing takes place once a year, to mitigate concerns of bias due to bid-ask spread bounces (see Core et al., 2008). AERS indicate the magnitude of the association between earnings quality measures and absolute excess returns. We use the magnitude of AERS as a proxy for the information content of an earnings quality measure.

We use this research design for all the earnings quality measures, which makes comparative evaluation possible. It also mitigates potential misspecification concerns in the hedge portfolio approach because, when comparing different measures, the respective measures act as controls of each other.

## 4. SAMPLE DESCRIPTION

The sample consists of US non-financial firms drawn from Compustat and CRSP over a 20-year period from 1988 to 2007. We do not use more recent data to avoid potential financial crisis effects. To analyze earnings quality measures over this period we require financial statements data from 1978 to 2008 because all the earnings quality measures are computed over a 10-year rolling estimation period, and some of them involve items over two or three consecutive periods. All accounting data are winsorized at the 1% level to control for outliers.

We require sufficient data to calculate all eight earnings quality measures for each firm in a yearly sample. To avoid excluding too many firms, we do not require data availability for each firm over the full 30-year period. As a consequence, the composition of firms in the yearly samples varies. Survivorship bias is expected to play a minor role in the analysis because it only arises for the 10-year estimation periods and the data requirements constrain the sample to more stable and long-lived firms. The total sample includes 27,589 firm–year observations, and the number of firms in each year varies between 1,265 and 1,509, with an average of 1,379. Table2 gives descriptive statistics of the main variables used to calculate the EQ measures and the controls over the 20 years.

**Table 2:** Descriptive Statistics of Main Variables

	*Mean*	*Std. Dev*.	*10%*	*25%*	*50%*	*75%*	*90%*
*NIBE*	0.0403	0.1379	−0.0383	0.0155	0.0485	0.0864	0.1317
*CFO*	0.0801	0.1601	−0.0247	0.0410	0.0880	0.1380	0.1956
*ACC*	−0.0398	0.0973	−0.1202	−0.0768	−0.0416	−0.0073	0.0409
*CACC*	0.0098	0.0939	−0.0603	−0.0204	0.0051	0.0360	0.0828
*PPE*	0.3839	0.2807	0.0908	0.1793	0.3153	0.5390	0.7815
Δ*REV*	−0.0141	0.5593	−0.2926	−0.1037	0.0000	0.0968	0.2519
Δ*AR*	−0.0026	0.0898	−0.0645	−0.0217	0.0000	0.0198	0.0565
*Assets*	6.1825	2.1005	3.4150	4.6323	6.1579	7.7135	9.0635
*Oper. cycle*	4.7474	0.6550	4.0113	4.4226	4.7958	5.1460	5.4609
*Intang. int*.	0.0861	2.5462	0.0000	0.0000	0.0000	0.0262	0.0773
*Capital int*.	0.3512	0.2318	0.0853	0.1686	0.2959	0.5002	0.7223
*Growth*	0.1231	1.6562	−0.1088	−0.0063	0.0640	0.1543	0.2988
*Leverage*	1.3254	35.5874	0.2648	0.5583	1.1298	1.9090	3.0102

This table reports the mean, the standard deviation, the 10^th^, 25^th^, 50^th^, 75^th^ and 90^th^ percentile for the main variables used. The sample period covers the period 1988–2007 and comprises 27,589 firm–year observations for which all the earnings quality measures under consideration can be computed. *NIBE*: net income before extraordinary items; *CFO*: cash flow from operations; *ACC*: total accruals; *CACC*: current accruals; *PPE*: gross property, plant and equipment; *ΔREV*: change in revenues; *ΔAR*: change in accounts receivable. All the above variables are scaled by total assets at the beginning of the period. *Assets*: natural logarithm of total assets; *operating cycle*: natural logarithm of the sum of days accounts receivable and days inventory; *intangible intensity*: reported R&D expense divided by sales (R&D expense is set to zero when absent); *capital intensity*: net book value of property, plant and equipment divided by total assets; *growth*: percentage change in sales; *leverage*: total liabilities divided by equity book value.

Table3 presents descriptive statistics for the eight EQ measures. Some measures are not symmetrically distributed, and some of the top and bottom deciles include extreme values. To mitigate the effect of potential outliers, we use quartiles to construct the hedge portfolios.

**Table 3:** Descriptive Statistics of Earnings Quality Measures

	*Mean*	*Std. Dev*.	*10%*	*25%*	*50%*	*75%*	*90%*
EQ1	0.3655	0.3660	−0.1037	0.1302	0.3832	0.6087	0.7839
EQ2	0.2390	0.2300	0.0066	0.0421	0.1676	0.3847	0.5915
EQ3	0.7094	0.3598	0.2858	0.4389	0.6731	0.9253	1.1602
EQ4	−0.6782	0.3235	−0.9673	−0.9171	−0.7924	−0.5470	−0.2036
EQ5	−0.0399	0.0545	−0.0924	−0.0508	−0.0242	−0.0100	−0.0037
EQ6	−0.0316	0.0286	−0.0652	−0.0397	−0.0235	−0.0135	−0.0077
EQ7	3.5770	5.3815	−0.5922	0.5723	2.2568	5.3645	9.7298
EQ8	0.2320	0.2087	0.0087	0.0525	0.1778	0.3657	0.5469

*Notes:* This table reports the mean, the standard deviation, the 10^th^, 25^th^, 50^th^, 75^th^ and 90^th^ percentiles for the eight earnings quality measures. Descriptions of the measures are given in Table1.

Table4 shows the Pearson correlations.^8^ With one exception, the correlations are significant, although most of them are economically small, which is consistent with results in prior literature such as, e.g., Francis et al. (2004). The correlations between the EQ measures are generally positive; there are few measures which are negatively associated with other measures, and the negative correlations are of smaller size than the positive ones. The generally low correlation suggests that the various measures capture different attributes or economic concepts. High correlations arise only between pairs of measures within the same set, particularly, +0.7519 for the time-series measures (EQ1 and EQ2), +0.8424 for smoothness (EQ3 and EQ4), only +0.4090 for accruals measures (EQ5 and EQ6), and +0.4923 for market-based measures (EQ7 and EQ8).

**Table 4:** Cross-Correlations of Earnings Quality Measures

	*EQ1*	*EQ2*	*EQ3*	*EQ4*	*EQ5*	*EQ6*	*EQ7*
EQ2	0.7519						
EQ3	0.0891	0.0584					
EQ4	0.0643	0.0367	0.8424				
EQ5	0.0276	0.0406	0.0163	0.0361			
EQ6	0.0922	0.1167	−0.3892	−0.2928	0.4090		
EQ7	0.1425	0.1757	−0.1543	−0.1630	0.0418	0.1621	
EQ8	0.0606	0.0491	−0.0884	−0.0959	*0.0051*	0.0576	0.4923

*Notes:* This table reports Pearson correlation coefficients between the 16 earnings quality measures. Most correlations are statistically significant at the 1% level; non-significant correlations are shown in italics.

Table5 shows the changes in the composition of the quartile portfolios based on the eight EQ measures over time. It shows the frequency of the annual changes of all firms across the different quartiles of EQ measures. No change is the most frequent result, with around 66% on average. A change from a low (high) EQ to a high (low) EQ portfolio occurs rarely. Portfolio selection based on abnormal accruals (EQ5) leads to the most changes in and outside the portfolios, selection based on accruals quality (EQ6) to the fewest changes. Even though the EQ measures are estimated on a rolling 10-year firm-specific basis, the portfolio allocation shows sufficient variation for further analyses, because the portfolios are based on ranks rather than absolute values of the measures.

**Table 5:** Frequency of Annual Changes across the Different Quartiles of Earnings Quality Measures

*(%)*	−*3Q*	−*2Q*	−*1Q*	*no change*	+*1Q*	+*2Q*	+*3Q*
EQ1	0.38	1.83	16.55	64.60	13.47	2.54	0.62
EQ2	0.38	2.85	16.03	62.38	15.37	2.57	0.41
EQ3	0.11	0.97	11.61	75.70	10.42	1.05	0.15
EQ4	0.12	0.92	11.84	75.11	10.87	1.03	0.12
EQ5	3.25	9.62	19.76	34.43	19.9	9.85	3.19
EQ6	0.02	0.52	8.31	81.10	9.48	0.54	0.02
EQ7	0.52	1.90	11.54	73.00	11.14	1.48	0.41
EQ8	0.70	3.03	15.38	61.44	16.05	2.90	0.50

*Notes:* This table presents the relative frequency of annual changes across the different quartiles (denoted by *Q*) of the earnings quality measures. The columns of the table refer to the quartile changes (for example, −3*Q* indicates the shift of a firm from the highest quartile to the lowest quartile of an earnings quality measure).

## 5. RESULTS

### (i) Ranking of Earnings Quality Measures

Table6 presents the main results. It shows the 1-year absolute excess returns of portfolios of firms with the highest and lowest values of the respective EQ measure, the absolute excess return spread (AERS), which is the difference between the value for the high-EQ portfolio and the low-EQ portfolio, and a significance test for the difference. The *t*-statistics are based on standard errors clustered by firm and year (following Petersen, 2009).

**Table 6:** Absolute Excess Returns by Earnings Quality Measures

	*High EQ*	*Low EQ*	*AERS*	*t-statistic*
EQ1	29.6173	32.8491	−3.2318	−4.3652***
EQ2	28.9981	32.6641	−3.6660	−6.1866***
EQ3	36.9001	27.4259	9.4741	7.9122***
EQ4	36.0349	27.6728	8.3621	7.3082***
EQ5	26.1654	38.8089	−12.6435	−10.2490***
EQ6	21.1395	43.1122	−21.9727	−12.8916***
EQ7	26.7328	35.1953	−8.4625	−7.5681***
EQ8	30.1621	31.6899	−1.5278	−1.7474*

*Notes:* This table presents 1-year mean absolute excess returns (as described in section 3.2) by earnings quality portfolio. High EQ refers to the top quartile portfolio of an earnings quality measure, low EQ to the bottom quartile portfolio. The absolute excess return spread (AERS) is computed as the difference between the mean value of the high-EQ portfolio and the mean value of the low-EQ portfolio. The return accumulation period starts 3 months after the end of the fiscal year and lasts 12 months. A *t*-test for the null hypothesis that the AERS is zero is reported (standard errors are clustered by firm and year). ***, ** and * indicate statistical significance at the 1%, 5% and 10% levels, respectively.

The first interesting observation is that the results are similar for the two EQ measures in each set of measures, although, as shown in Table4, most of the cross-correlations are relatively low. This suggests that the measures in each set capture the same construct according to AERS. A high correlation is not a necessary condition for measures to serve as proxies for the same underlying concept.

Hypothesis H1 predicts a negative association between the value of an earnings quality measure and the absolute value of excess returns. We test this hypothesis by considering the sign of the AERS, which under H1 should be negative.

Table6 shows that all eight EQ measures yield significant AERS. As predicted, the AERS are negative for time-series measures, accruals measures and market-based measures. This is consistent with the prediction that these measures help to reduce mispricing errors. However, AERS are significantly positive for the smoothness measures. This result suggests that smoothness reflects useful information for pricing firms, hence, more smoothness should be interpreted as better earnings quality on average. While accruals measures also allow for different interpretations, our results support the common view that higher values of our accruals measures EQ5 and EQ6 indicate higher earnings quality.

To test hypothesis H2, we use the unsigned magnitude of AERS as a proxy for the information content of the earnings quality measures. The absolute value of AERS is highest for EQ6 (accruals quality), which therefore is most successful in distinguishing between the two portfolios. EQ5 (abnormal accruals) comes next, followed by EQ3 (smoothness), EQ7 (earnings response coefficient), and EQ4 (smoothness). The two time-series measures show low AERS, and the lowest AERS is for EQ8 (value relevance). Table7 shows the differences in magnitude of AERS and a test statistic for these differences, which are calculated from a two-sided *z*-test using the difference of the values divided by its standard error. The standard errors are obtained from a bootstrapping procedure with 1,000 replications. Accruals measures display the highest magnitude of AERS; specifically, the AERS associated with accruals measures are significantly greater than the AERS associated with the other measures.

**Table 7:** Absolute Excess Return Spread Differences across Earnings Quality Measures

	*EQ1*	*EQ2*	*EQ3*	*EQ4*	*EQ5*	*EQ6*	*EQ7*
EQ2	−0.4342						
EQ3	−6.2423***	−5.8081***					
EQ4	−5.1303***	−4.6961***	1.1120				
EQ5	−9.4117***	−8.9775***	−3.1694***	−4.2814***			
EQ6	−18.7409***	−18.3067***	−12.4986***	−13.6106***	−9.3292***		
EQ7	−5.2307***	−4.7965***	1.0116	−0.1004	4.1810***	13.5102***	
EQ8	1.7040*	2.1382*	7.9463***	6.8343***	11.1157***	20.4449***	6.9347***

*Notes:* This table reports the difference in the absolute excess return spread (AERS) across the 16 earnings quality measures. Differences are calculated as the absolute value of the AERS of the column EQ minus the absolute value of the AERS of the row EQ as reported in Table6. Significance is computed by a *z*−test for the null hypothesis that the difference in AERS is zero, based on bootstrapped standard errors. ***, ** and * indicate statistical significance at the 1%, 5% and 10% levels, respectively.

These results provide support for the claim made in the empirical literature that accruals measures, and particularly accruals quality, are preferable proxies for earnings quality. In our hypothesis development, we noted that measures that are based on a broader set of information are likely to be of higher quality than other measures. Abnormal accruals (EQ5) use the largest set of financial items, and this can increase their ability to discriminate excess returns. A possible reason for the high performance of accruals quality (EQ6) could be that it is the only measure that uses forward information (cash flow from operations of the subsequent period), which should give it an unfair comparative advantage. We also calculate the AERS lagged by one period and, as expected, the magnitude slightly decreases, to –21.2105; however it is still the largest magnitude.^9^

Smoothness measures process information on the volatility or the correlation of earnings, cash flows and accruals. Time-series and value relevance measures use only net income from the financial statements, which is likely to reduce their quality. Time-series measures are based only on accounting earnings, although they track their evolution. On the other hand, market-based measures are the only measures that incorporate market returns, which should give them a relative advantage over time-series measures. The two market-based measures result from the same econometric regression, and it is surprising that the earnings response coefficient yields a high magnitude of AERS, whereas value relevance (EQ8) does worst among the eight measures we consider. As noted earlier, accruals measures and smoothness measures may be subject to different interpretations.

We next examine how the EQ measures interact with one another in the determination of AERS, in order to assess whether an EQ measure preempts the AERS of other measures and hence is a stronger measure empirically. We take a double-sorting approach as, for example, in Landsman et al. (2011). For each pair of measures EQ*_i_* and EQ*_j_* and for each year, we first group firms in four portfolios based on EQ*_i_* and rank the firms in each of the quartile portfolios based on EQ*_j_*. We then pool all observations in the four groups and compute the AERS corresponding to EQ*_j_*. The results, presented in Table8, are generally consistent with the signs and the significance levels of AERS in the main analysis. The significance levels decline with an increase in the correlation between pairs of measures. In sum, the results suggest that possible interactions among the EQ measures do not affect our main findings, and the measures measure somewhat different constructs.

**Table 8:** Controlling for the Interaction between Earnings Quality Measures

	*EQ1*	*EQ2*	*EQ3*	*EQ4*	*EQ5*	*EQ6*	*EQ7*	*EQ8*
EQ1	–	−0.9775	−3.6412***	−3.8707***	−2.9724***	−1.9784***	−1.4978**	−3.2801***
EQ2	−1.9329**	–	−3.8618***	−3.8072***	−3.2142***	−2.2041***	−1.9770***	−3.6467***
EQ3	9.7508***	9.8369***	–	4.3277***	9.8571***	1.9012*	7.2109***	9.3557***
EQ4	8.8683***	8.6630***	1.1379**	–	9.2003***	1.5658*	6.2952***	8.4208***
EQ5	−12.4153***	−12.5724***	−12.6378***	−12.9286***	–	−5.3235***	−12.0591***	−12.6724***
EQ6	−21.8231***	−21.6975***	−20.0592***	−20.4408***	−18.3326***	–	−20.3980***	−21.7998***
EQ7	−7.7766***	−7.7448***	−6.2087***	−6.4157***	−7.7431***	−4.2119***	–	−9.0382***
EQ8	−1.5143*	−1.3512	−0.8814	−0.8257	−1.8191**	−0.5384	5.4124***	–

*Notes:* This table presents 1-year mean absolute excess returns (AERS, as described in section 3.2) obtained after controlling for the interaction between pairs of earnings quality measures. Specifically, for each pair of measures *i* and *j* and for each year, we first group firms in four portfolios based on *i*, then we rank the firms in quartile portfolios based on *j*; we pool all the observations and compute AERS as the mean difference between absolute excess returns in the top and in the bottom quartile portfolios of *j*. The columns indicate the measures by which firms are sorted first; the rows show the measures for which AERS returns are then computed. A *t*-test for the null hypothesis that the AERS is zero is reported (standard errors are clustered by firm and year). ***, ** and * indicate statistical significance at the 1%, 5% and 10% levels, respectively.

We also examine the association between AERS and the four sets of EQ measures. We combine the measures into time-series measures (EQ1, EQ2); smoothness measures (EQ3, EQ4); accruals measures (EQ5, EQ6); and market-based measures (EQ7, EQ8). For each year we rank firms in deciles corresponding to each measure, and then obtain the index as the average rank across the measures considered. An analogous approach to aggregating different measures is used, for example, by Bhattacharya et al. (2003) to derive an earnings opacity measure. Consistent with the main findings, the results show that time-series, accruals and value relevance measures yield negative and significant AERS, while smoothness measures yield positive and significant AERS.

The EQ measures may be affected by the business model, operating risk and the operating environment (Francis et al., 2006 refer to these as innate factors).^10^ Innate factors are likely to affect the earnings quality measures differently. For example, the two time-series measures are likely to depend more strongly on innate factors than abnormal accruals and market-based measures.

To test whether the results are affected by innate factors, we perform a similar analysis with residual form measures. Based on the literature, we use the following controls: *assets*: natural logarithm of total assets; *operating cycle*: natural logarithm of the sum of days accounts receivable and days inventory;^11^ *intangible intensity*: reported R&D expense divided by sales (R&D expense is set to zero when absent); *capital intensity*: net book value of property, plant and equipment divided by total assets; *growth*: percentage change in sales; *leverage*: total liabilities divided by equity book value. The residual form measures are the residuals of yearly regressions of the EQ measures on the innate factors.

Table9 presents the results. As expected, the amounts of AERS are smaller than those for the original measures, but the ranking is basically similar. A notable difference is that, in residual form, EQ5 yields lower AERS than market-based measures. In general, however, we conclude that differences in the operating environment are not a major driver of our results.

**Table 9:** Residual-form Measures

	*High EQ*	*Low EQ*	*AERS*	*t-statistic*
EQ1^R^	29.5317	31.7071	−2.1754	−2.9936***
EQ2^R^	29.0789	30.3011	−1.2222	−2.0733**
EQ3^R^	34.2000	28.5045	5.6955	5.2717***
EQ4^R^	34.0831	29.4960	4.5872	4.4541***
EQ5^R^	32.2966	35.0003	−2.7037	−3.0339***
EQ6^R^	28.5767	38.6125	−10.0358	−7.5496***
EQ7^R^	26.4337	32.1767	−5.7431	−7.8016***
EQ8^R^	28.9900	32.3737	−3.3838	−4.3757***

*Notes:* This table presents 1-year mean absolute excess returns (as described in section 3.2) by earnings quality portfolio. EQ^R^ are the residual measures obtained after controlling for innate factors. High EQ refers to the top quartile portfolio of an earnings quality measure, low EQ to the bottom quartile portfolio. The absolute excess return spread (AERS) is computed as the difference between the mean value of high-EQ portfolio and the mean value of the low-EQ portfolio. The return accumulation period starts 3 months after the end of the fiscal year and lasts 12 months. A *t*-test for the null hypothesis that the AERS is zero is reported (standard errors are clustered by firm and year). ***, ** and * indicate statistical significance at the 1%, 5% and 10% levels, respectively.

### (ii) Alternative Explanations

In this section we address alternative explanations for excess returns. First, we examine how information uncertainty and the environment affect our results, second, how they are related to known pricing anomalies and third, whether the expected return model affects our results.

As we discuss in the hypotheses development, stock prices aggregate financial and other information. In particular, poor earnings quality increases the information uncertainty, which may induce investors and financial intermediaries to search for more information, which is then reflected in the stock price and helps reduce mispricing. That is, earnings quality can affect the information environment in a systematic way.^12^ We consider information uncertainty using two sets of variables: fundamental volatility (volatility of operating profitability and volatility of operating cash flows) and analyst-related variables (analyst coverage and forecast dispersion). Such variables have been used in prior literature (e.g., Zhang, 2006; Burgstahler and Chuk, 2010). Other possible differences in the information environment are captured by innate factors that we test above.

We measure fundamental volatility as the standard deviation of net income before extraordinary items (*NIBE*), and of cash flow of operations (*CFO*), each scaled by prior year total assets over a rolling 10-year period from *t*–9 to *t*.

Analyst coverage is calculated as the natural logarithm of 1 plus the number of analysts following at the end of the fiscal year; analysts’ forecast dispersion is the standard deviation of analysts’ forecasts on earnings per share at the end of the fiscal year, divided by the stock price at the end of the fiscal year. Data requirements reduce the sample size for tests based on analyst-related variables to 16,150 and 13,767 firm–year observations when using analyst coverage and forecast dispersion, respectively.

We also consider firm size, measured as the natural logarithm of market value of equity at the end of the fiscal year. Size is often used as an additional measure of information uncertainty, because larger firms are expected to have a richer information environment. We note, however, that size is a proxy for a multitude of factors; furthermore, it is partly controlled for in the four-factor expected returns model, although only at a portfolio level.

We again take a double-sorting approach. For each year, we group firms in three portfolios based on the magnitude of each of these five variables. Then we rank the firms in each of the portfolios on the respective EQ measure. We pool firms in the top and bottom quartiles over the three portfolios and all years and calculate the AERS for these portfolios. Table10 presents the results. The magnitudes of AERS are somewhat smaller, but the order is qualitatively the same. A significant difference occurs for smoothness measures in the double-sorting by volatility of *NIBE*; since smoothness is essentially based on the same measure, this result is not relevant. Another difference is that the AERS corresponding to EQ1 is not significantly different from zero in the double-sorting by forecast dispersion.

**Table 10:** Controlling for Information Uncertainty

	*Earnings Volatility*	*Cash Flow Volatility*	*Size*	*Analyst Coverage*	*Forecast Dispersion*
EQ1	−3.0636***	−1.9596***	−1.5462**	−2.4279***	−0.9831
EQ2	−3.2511***	−2.0686***	−1.7459***	−2.8016***	−1.7584***
EQ3	−1.6261*	9.8131***	7.7330***	6.7064***	4.2274***
EQ4	−2.9353***	8.3670***	7.1777***	6.2182***	3.9526***
EQ5	−6.6943***	−5.1799***	−7.8905***	−6.7733***	−6.9638***
EQ6	−10.0891***	−12.4444***	−15.1780***	−16.8334***	−13.6463***
EQ7	−3.0481**	−5.6053***	−5.5703***	−5.5710***	−3.2940***
EQ8	−0.4777	−1.8358**	−2.6964***	−1.2980*	−0.7318

*Notes:* This table presents absolute excess return spreads (AERS, as described in section 3.2) by earnings quality portfolio. For each fiscal year, firms are first assigned to three portfolios based on the magnitude of: volatility of net income before extraordinary items (standard deviation of *NIBE*, scaled by prior year total assets, over years *t*–9 to *t*), volatility of cash flow from operations (standard deviation of *CFO*, scaled by prior year total assets, over years *t*–9 to *t*), size (natural logarithm of market value at the end of the fiscal year), analyst coverage (natural logarithm of 1+number of analysts following at the end of the fiscal year), forecast dispersion (standard deviation of analysts’ forecasts on earnings per share at the end of the fiscal year, divided by the stock price at the end of the fiscal year). Within each portfolio, firms are assigned to the earnings quality portfolios. The return accumulation period starts 3 months after the end of the fiscal year and lasts 12 months. A *t-*test for the null hypothesis that the AERS is zero is reported (standard errors are clustered by firm and year). ***, ** and * indicate statistical significance at the 1%, 5% and 10% levels, respectively.

A second set of tests addresses the question of whether our earnings quality results merely reflect pricing anomalies identified in prior research.^13^ We consider four well-documented anomalies: price momentum (Jegadeesh and Titman, 1993), asset growth (Fairfiel et al., 2003), book-to-market ratio (Fama and French, 1992) and accruals (Sloan, 1996).^14^ Momentum is measured as the cumulative stock return from month *t*–12 to month *t*–2, where *t* is the month in which excess returns begin to accumulate; asset growth is measured as the natural logarithm of the assets at fiscal year end divided by assets at the previous fiscal year end. The book-to-market ratio is measured as the natural logarithm of the ratio of equity book value to market value of equity. Finally, accruals (*ACC*) are used to capture the accrual anomaly. Using a similar double-sorting approach for each of these variables (untabulated) produces qualitatively similar results.

In untabulated tests, we double-sort firms based on trading volume of the stock, using share turnover (logarithm of the number of shares traded divided by the number of shares outstanding during the year) as proxy. In addition, double-sorting based on illiquidity, using the daily absolute stock return per dollar trading volume (Amihud, 2002) averaged over the fiscal year corresponding to the EQ measure, yields similar results. We also perform the analysis after excluding penny stocks (stock price less than one dollar);^15^ the results are essentially unchanged. Overall, these tests suggest that the results are not likely to be driven by common pricing anomalies. The variables used in the double-sorting tests are also related to potential other differences between firms in the two EQ portfolios. Given the results, structural differences in the composition of the portfolios are unlikely to exist.

Our third set of tests considers the expected returns. Errors in estimated expected returns can show up as excess returns, because excess returns are calculated as the difference between actual returns and expected returns. In line with the broad literature, we use the Fama–French three factors and momentum and estimate the factor loadings over 36 months for each firm. It is well known that firm-specific estimates are subject to low power and vary over time, which may confound our results.

There is no consensus regarding which model is the best to estimate expected returns and which factors constitute pricing anomalies (see, for example, Kothari et al., 1995; MacKinlay, 1995; Daniel and Titman, 1997). To examine whether our results depend on the expected returns model, we calculate excess returns based on one factor, market risk, and on the three Fama and French factors without momentum. The results, which are presented in Table11, are qualitatively unchanged.

**Table 11:** One-factor and Three-factor Models for Expected Returns

	*High EQ*	*Low EQ*	*AERS*	*t-statistic*
**Panel A: One-factor Model**				
EQ1	29.8538	32.5806	−2.7268	−3.8317***
EQ2	29.0767	32.6148	−3.5381	−5.8993***
EQ3	36.1682	27.5371	8.6311	7.5230***
EQ4	35.4990	27.8236	7.6754	7.4626***
EQ5	26.2773	38.0583	−11.7810	−8.6989***
EQ6	21.6801	42.1014	−20.4213	−11.8661***
EQ7	26.6834	35.2508	−8.5674	−6.2163***
EQ8	30.1167	31.7824	−1.6657	−2.0088**
**Panel B: Three-factor Model**				
EQ1	29.3328	32.3034	−2.9706	−4.0172***
EQ2	28.7464	32.2137	−3.4673	−5.9047***
EQ3	36.4064	27.1287	9.2777	7.5208***
EQ4	35.5659	27.3739	8.1920	7.1636***
EQ5	25.9374	38.1181	−12.1807	−10.0877***
EQ6	20.9765	42.4195	−21.4429	−12.6386***
EQ7	26.4087	34.8689	−8.4602	−7.4088***
EQ8	29.6394	31.4446	−1.8052	−2.1323**

*Notes:* This table presents 1-year mean absolute excess returns by earnings quality portfolio. Excess returns are obtained by using a one-factor model (Panel A: the only risk factor is the market return) or a three-factor model (Panel B: the risk factors are the market return, book-to-market and size). *High EQ* refers to the top quartile portfolio of an earnings quality measure, *low EQ* to the bottom quartile portfolio. The absolute excess return spread (AERS) is computed as the difference between the mean value of high-EQ portfolio and the mean value of the low-EQ portfolio. The return accumulation period starts 3 months after the end of the fiscal year and lasts 12 months. A *t-*test for the null hypothesis that the AERS is zero is reported (standard errors are clustered by firm and year). ***, ** and * indicate statistical significance at the 1%, 5% and 10% levels, respectively.

Finally, earnings quality can be correlated with the expected cost of equity capital.^16^ It is moot whether earnings quality (or, more generally, information quality) is a priced risk factor, or not. For example, Francis et al. (2005) suggest that accruals quality is a priced risk factor, capturing non-diversifiable information risk. In contrast, Core et al. (2008) find no support for the conclusion that accruals quality is a priced risk factor. We note that if there is a correlation between earnings quality and expected returns, it affects the calculation of excess returns. However, we have no priors for how such a correlation could impact our results, as the mechanism underlying market mispricing is different.

### (iii) Sensitivity Tests

We run a number of sensitivity checks to assess the robustness of our results; the results of these tests are untabulated for brevity. We compute the returns in the market-based measures for 15-month windows rather than 12-month windows (beginning 3 months after the fiscal year end). Furthermore, we repeat the analysis with 30% top and bottom portfolios instead of 25%. To alleviate the potential concern that the loadings of the expected returns model are not stable over time (e.g., Fama and French, 2007), we reestimate the model using a 24-month and a 48-month estimation period. Since raw data on cash flows from operations are not available for the early periods, we calculate *CFO* following the literature indirectly by adjusting net income for changes in certain balance sheet items. Alternatively, we use the reported cash flows from operations for the periods for which they are available. In all these tests, we find no qualitatively different results.

To address potential concerns that the results are driven by the period for which we collect data,^17^ we run the tests with shorter periods of the first 10 years (1988 to 1997) and the last 10 years (1998 to 2007) and find results that are qualitatively similar to those reported in Table6 for the 20 years; the only exception is EQ8, with an insignificant AERS in the first 10 years.

The empirical design we employ focuses on 1-year excess returns, beginning 3 months after fiscal year end. Contemporaneous returns may be better in capturing systematic risk. We modify the accumulation period from months *t*–8 to *t*+3, that is, when the financial statements are assumed to become available. The results are qualitatively similar; the only exception is EQ8, which now displays insignificant AERS. This further mitigates concerns about the instability of loadings over time. Landsman et al. (2011), among others, argue that mispricing is more a short-term effect and should diminish over time, whereas model errors are likely to be stable. We therefore calculate 2-year and 3-year AERS and find that AERS tend to diminish in the third year. This supports the mispricing interpretation of the results.

Prior literature suggests that the usefulness of earnings quality measures is different for profit-making and loss-making firms because, for example, losses are less persistent than profits. The earnings response coefficient may also differ for losses and gains due to conservatism (Basu, 1997). Balakrishnan et al. (2010) argue that investors may not fully understand the different persistence of losses. To see how these considerations affect our results, we split the sample into firms with positive and negative earnings (*NIBE*) and calculate AERS. The results are qualitatively similar for loss-making and profit-making firms, except that EQ8 has insignificant AERS in both sub-samples; furthermore, for the loss-making firms, EQ3 and EQ4 have AERS significant only at the 5% and 10% levels, respectively. We also double-sort firms based on profitability (following Fama and French, 2008), using the ratio of equity income over equity book value as a measure of profitability, and find qualitatively similar results to the main findings.

While our focus is on absolute excess returns, we also examine the signed excess returns. The results show that significantly negative hedge returns can be obtained by trading on time-series and accruals measures, whereas market-based measures yield significantly positive hedge returns.^18^ The results on hedge returns differ substantially from our main results. However, the hedge returns are below 3%, which is economically not large.^19^ This suggests that excess returns are not directed towards either underpricing or overpricing, supporting our prediction.

## 6. CONCLUSIONS

Despite the extensive use of earnings quality measures in the literature, little is known about which measures are good proxies for unobservable, true earnings quality. This paper contributes to a better understanding of the quality of earnings quality measures by examining their association with absolute excess returns, which are a proxy for stock mispricing due to low-quality information. We study eight different measures: six accounting-based measures (persistence, predictability, two measures of smoothness, abnormal accruals and accruals quality) and two market-based measures (earnings response coefficient and value relevance).

We form portfolios based on each earnings quality measure and calculate the difference in the mean absolute excess returns for the high-quality and the low-quality portfolios. Using a large sample of US non-financial firms over the period 1988–2007, we first confirm that time-series measures, accruals measures (multiplied by –1) and market-based measures are negatively associated with the absolute value of excess returns. In contrast to the prevailing findings, smoothness measures are positively associated with absolute excess returns, which suggests that higher smoothness captures higher earnings quality. Second, we find that accruals measures are the most useful earnings quality measures, as measured by the reduction in the absolute excess returns earned for the hedge portfolios. This finding provides support for the broad use of these measures in empirical studies of earnings quality.

There are several caveats to our study. While the predicted relationship between earnings quality and absolute excess returns is intuitive, there may be other reasons for this relationship than mispricing. For example, both can be affected by the operating environment and information uncertainty. While we control for these effects to some extent, we cannot exclude the possibility that they influence our results. Moreover, calculating excess returns from the difference between actual and expected returns requires the use of a model to estimate expected returns: there is no consensus on what a good model is, and whether earnings quality is a priced risk factor. Future research can help shed more light on these relationships.

## APPENDIX

### Variables Definition

**Table:** 

***Earnings Quality Measures***	
*NIBE*	Net income before extraordinary items (Compustat IB).
*CFO*	Cash flow from operations: CFO = NIBE – ACC.
*ACC*	Total accruals: *ACC* = Δ*CA* – Δ*CL* – Δ*CASH* + Δ*STDEBT* – *DEPR*. Δ*CA* is change in current assets (Compustat ACT), Δ*CL* is change in current liabilities (Compustat LCT), Δ*CASH* is change in cash (Compustat CHE), Δ*STDEBT* is change in short-term debt (Compustat DLC), *DEPR* is depreciation (Compustat DP).
*CACC*	Current accruals: the sum of total accruals (*ACC*) and depreciation (Compustat DP).
*PPE*	Gross property, plant and equipment (Compustat PPEGT).
Δ*REV*	Change in revenues (Compustat REVT).
Δ*AR*	Change in accounts receivables (Compustat RECT).
*RET*	12-month stock return (source CRSP) ending 3 months after the end of the fiscal year.
*P*	Market value of equity (Compustat CSHO times Compustat PRCC_F) at the beginning of the fiscal year.
***Innate Factors of Earnings Quality***	
*Assets*	Natural logarithm of total assets (Compustat AT).
*Operating cycle*	Natural logarithm of the sum of days accounts receivable (365 divided by the ratio of Compustat SALE to Compustat RECT) and days inventory (365 divided by the ratio of Compustat COGS to Compustat INVT).
*Intangible intensity*	Reported R&D expense (Compustat XRD) divided by sales (Compustat SALE). R&D expense is set to zero when absent.
*Capital intensity*	Net property, plant and equipment (Compustat PPENT) divided by total assets (Compustat AT).
*Growth*	Percentage change in sales (Compustat SALE).
*Leverage*	Total liabilities (Compustat LT) divided by equity book value (Compustat CEQ).
***Excess Returns***	
*R*_*i*_	Actual monthly return of firm *i* (source CRSP).
*R*_*f*_	Monthly riskless rate of return (source Kenneth French's website), measured as the 1-month Treasury bill rate.
*R*_*M*_	Monthly market return (source Kenneth French's website), measured as the value-weighted return on all NYSE, AMEX, and NASDAQ stocks.
*SMB*	Monthly return on the size factor mimicking portfolio (source Kenneth French's website).
*HML*	Monthly return on the book-to-market factor mimicking portfolio (source Kenneth French's website).
*UMD*	Monthly return on the momentum factor mimicking portfolio (source Kenneth French's website).
***Information Uncertainty***	
*Earnings volatility*	Standard deviation of *NIBE*, standardized by prior year total assets (Compustat AT), from year *t*–9 to year *t*.
*Cash flow volatility*	Standard deviation of CFO, standardized by prior year total assets (Compustat AT), from year *t*–9 to year *t*.
*Analyst coverage*	Natural logarithm of (1 + number of analysts following at the end of the fiscal year) (source I/B/E/S).
*Forecast dispersion*	Standard deviation of analysts’ forecasts of earnings per share at the end of the fiscal year, divided by the stock price at the end of the fiscal year (source I/B/E/S).
*Size*	Natural logarithm of market value of equity at the end of the fiscal year (Compustat CSHO times Compustat PRCC_F).

## Footnotes

1 Lee and Liu (2011) survey the different views on the relationship between idiosyncratic volatility and the informativeness of stock prices.

2 While the results presented below are in line with Francis et al. (2004) for accruals quality, the ranking of their other measures differs from ours.

3 This specification follows the modification of Dechow et al. (1995).

4 Francis et al. (2005, p. 299) suggest taking the absolute values for an EQ measure and signed accruals for studying earnings management.

5 Risk free rates, the market returns, and the portfolio factor returns *SMB*, *HML* and *UMD* are obtained from French's website (http://mba.tuck.dartmouth.edu/pages/faculty/ken.french/data_library.html).

6 An alternative approach is to use linear regression analysis of absolute excess returns on the EQ measures. For example, Fama and French (2008) use both the sorting approach and the regression approach for a number of pricing anomalies and obtain analogous results; they also discuss the advantages and disadvantages of the two methods. We do not have priors for the linearity of excess returns based on earnings quality measures, and we use the hedge portfolio approach because it is more robust to deviations from linearity and less sensitive to outliers.

7 A potential disadvantage is a lower significance of the return differences across portfolios. This biases our results against revealing significant effects.

8 Spearman correlations are similar to Pearson correlations. Furthermore, correlations in the stocks in the extreme EQ quartile portfolios are similar.

9 Since we are interested in comparing the quality of the earnings quality measures as they are used in the literature, we retain the original forward information definition of EQ6 in subsequent analyses.

10 Another potential factor is the reporting environment, which includes accounting standards and their application, management incentives, auditing, corporate governance, and enforcement. We believe that these factors are not of first-order relevance in our sample.

11 The days accounts receivable are calculated from 365 times accounts receivables turnover over sales; the days inventory from 365 times inventory over cost of goods sold.

12 See, for example, Daniel et al. (1998,^2001^), who find that return predictability increases with information uncertainty.

13 See, for example, Green et al. (2013) for a comprehensive list of factors that have been found to affect stock returns.

14 We note that the four-factor model we use to calculate expected returns captures several such factors, although on a portfolio rather than firm-specific basis.

15 Penny stocks are often found to be related to mispricing anomalies. For example, Kim and Qi (2010) find that the pricing effect of accruals quality is not present in low priced stocks.

16 See, for example, Easley and O'Hara (2004), Hughes et al. (2007), Yee (2006) and Lambert et al. (2007).

17 See, for example, Core et al. (2008) who find significant differences for different periods.

18 Using double-clustered standard errors, only the hedge returns on EQ1, EQ2 and EQ8 are significantly different from zero.

19 Green et al. (2013) report significantly larger hedge returns for other factors.
